# Hospital and Institutional News

**Published:** 1918-10-05

**Authors:** 


### HOSPITAL AND INSTITUTIONAL NEWS.

### OCTOBER 12, 1918: ABSIT OMEN.

In the *Hospital Saturday Fund Journal* dated September 30 appears a strong appeal to theatre proprietors and their patrons not to withdraw support from the metropolitan hospitals on "Hospital Saturday," October 12. The risk is real, because collections will be made on behalf of "Our Day" at many theatres, music-halls, cinemas, at every performance taking place between Monday, September 30, and October 24, inclusive. If this be done, it will shut out the metropolitan hospitals on "Hospital Saturday," October 12—a procedure which will neither be just to the hospitals nor to be desired by the British Red Cross Society. It is unfortunate that the Entertainments Industry Committee, representing theatrical proprietors and their musical and histrionic staffs, did not consider, when organising their great united effort for "Our Day," what, if any, classic collections were annually due during the same period. Hospital Saturday is such a classic anniversary, consecrated to this season, and the theatrical profession has identified itself so closely with hospital interests, that the oversight in the arrangements is a strange blunder. To avoid upsetting either the old organisation or the new, it would be necessary only, in our view, to make the collections daily as announced, but on Hospital Saturday to hold them, by announcement, for the benefit of the Hospital Saturday Fund. If a simpler plan and one more advantageous to both parties can be suggested, we should be very glad to hear of it at once.

### SHORTENING THE MEDICAL CURRICULUM.

On October 2 Dr. Addison, Minister of Reconstruction, gave an important address to the School of Pharmacy of the Pharmaceutical Society (see page 13 of our present issue). In passing, it may be mentioned that Wednesday of this week was a red-letter day in the history of the Pharmaceutical Society, for never before, we believe, since its foundation three-quarters of a century ago has it been honoured by a visit of a Cabinet Minister to its headquarters on an official occasion. Moreover, Dr. Addison was supported by a number of high officials, including Colonel Lord Gorell, of the Department of the Directorate of Staff Duties in the Army, Sir W. H. Norman, C.B., Director-General of the Medical Department of the Navy, and General Goodwin, C.M.G., D.S.O., Director-General of the Army Medical Service. Incidentally, the presence of these officers suggests the excellent relationship that exists between medicine and pharmacy.

### THE NATURAL LIMITS OF DEMOCRACY.

The size of committees to which Labour representatives are added in proportion to the number of their electors or the amount subscribed by them is apt to be unwieldy, with the result in time that a smaller executive within the managing body has to arise if the work is to be satisfactorily carried on. When this happens, there is always the risk of some irresponsible talk of "a cabinet," a "bureaucracy," an "inner ring," or a "gang," to express the awakening of the sense that even representative institutions have, like all others, their inherent limitations on efficiency. Though it is a good thing to have doctrinaire democrats brought to their senses by such a return to realities as this cry reveals, it is never a very pleasant time for the hospital in connection with which the awakening occurs. An instance in point is that of the Swansea Hospital. Here the existence of "a cabinet," in the form of a, relatively, small house committee has been discovered. The Labour members on the board demand that the representatives of the works on the board of management should be increased from eighteen to twenty-four, and that they should have eight seats instead of four upon the "cabinet" or house committee. The board is usually attended by forty members, and since Labour members seem to have more time to waste on big committees than their less leisured colleagues, it is presumed that twenty-four seats will give them a majority on the board. The house committee, we mean the "cabinet," consists of sixteen members. Thus the proposal of the Labour members, if acted on, will either give them half the seats, or increase the "small" committee to twenty! The latter proposal will prove the worse, unless there is sufficient good sense on the part of several members to absent themselves habitually from a committee whose efficiency is exactly proportioned to the smallness of its size. When will people realise that though representative institutions may be big, executives must be small? The whole thing will only end in the creation of "an inner cabinet," which will be not only a *reductio ad absurdum*, but also a return to common sense.

### A BROKEN PLEDGE AND ITS CONSEQUENCE.

A curious case of an apparently broken pledge, for which a medical superintendent has been called to order, has occurred in connection with the treatment of soldiers in Greenwich Union Infirmary, now the Greenwich Metropolitan Auxiliary Hospital. The case arose out of a correspondence between Colonel R. J. S. Simpson, A.D.M.S., Woolwich District, and Dr. Wiggins, the medical superintendent, wherein the latter's arrangements for the medical supervision of the military patients in the infirmary were called in question. At an extraordinary meeting of the Guardians held to consider the controversy, Mr. J. E. Morton, chairman of the Infirmary Committee, stated that Dr. Wiggins had given a pledge to Colonel Simpson that the medical supervision of the military patients should be thorough and effective, but that the chairman of the Board, himself (Mr. Morton) and the Clerk had discovered that the pledge had not been observed. Dr. Wiggins had then been informed that the pledge must be fulfilled. Mr. Morton received the support of the Board in the action which he had taken.

### AN ADVENTUROUS MEDICAL CAREER AT HOME AND ABROAD.

Dr. Beale Collins, for twenty-five years Medical Officer of Health for Kingston, has died suddenly of heart failure at the age of sixty-nine. His career was adventurous to the end. In 1873 he was instructor in naval hygiene at Haslar. Later he joined the Perak Expedition, saw the end of the Russo-Turkish War, served in China, travelled in Roumania, up the Danube, in the Dardanelles, and retired in 1884 to engage in public practice at home. By 1902 he had become president of the Society of Medical Officers of Health. After taking on extra medical war work a few months ago he broke down, went to the sanatorium at Midhurst, then to the Isle of Wight, where he recovered so well that he took a house near Ventnor. Two days after joining his family there, he died in the vigour of age and energies.

### THE AMERICANS AT MOSSLEY HILL.

Mossley Hill, the American Red Cross Hospital at Liverpool, has a special interest in that it was the first hospital for American soldiers to be opened in this country. We may pride ourselves that it was the gift of Dr. Edmund Muspratt, of Liverpool, who offered his house for the nominal rent of £40 per annum. It was officially opened on January 11, when it became the American Red Cross Military Hospital No. 4. Its seventy-five beds were under the charge of Major Udo J. White, formerly Professor of Surgery at Michigan University. It has since undergone a series of enlargements. Its first seventy-five beds were quickly increased to 200, then to 500; then in April reconstruction was required. The staff jumped from three officers and twelve nurses to twelve and thirty respectively, and, apart from a projected number of 150 enlisted men, the new scheme will increase the staff to eighteen officers and sixty nurses under Captain M. W. Leonard, of New York. Of the reconstruction, which has changed the hospital from one for special cases to a distributing centre for England, two wards remain to be completed, but 300 further patients can be accommodated in the carefully provided tents which have been prepared. Mossley House itself is now the administrative block in the centre of a score of buildings, which include nine wards, a recreation-hut, a dining-hall, and an extensive Red Cross Club for nurses.

### THE SITE OF BATTLE.

Monmouthshire is to have another isolation hospital, to be jointly used by the six urban districts and one rural district of the Eastern Valleys stretching from Blaenavon to Llantarnam, a name made famous by W. H. Davies, the poet. There is somewhat of a deadlock, however, as to its locality; indeed, there has been quite a battle over the site question. To arrive at a decision a commission from the Monmouthshire County Council held an inquiry at Pontypool on September 26, when it was reported that Dr. Rocyn Jones, the county medical officer, suggested that the most suitable sites for a new building were either near Trevethin, Pontypool, or just below Pontypool Road. A number of the representatives of the bodies concerned considered that the latter site was the more central and accessible, and it was generally agreed also that the County Council should bear most, if not the whole, of the capital and administrative cost.

### FROM FIRE DRILL TO FUEL DRILL.

Coal-saving now ranks as an important patriotic duty. Institutions should give a good lead to the public in the matter of fuel economy. Much, of course, depends on the efforts of individual members of the staff, therefore the example might profitably be followed of the medical officer of the Mental Hospital at Hanwell, who is specially instructing the staff on how fuel and lighting may be saved. Hospitals may, moreover, make their influence felt in a wider sphere still, if they adopt the system now in vogue at the London Hospital. There every patient on leaving is given a pamphlet which shows how coal and gas can be saved, and not only are detailed instructions in a like sense given to each nurse in the institution, but everyone sent out to a private case acts also as a missionary in the coal-saving campaign.

### Y.M.C.A. AND AN AWKWARD DILEMMA.

Southwark Board of Guardians have evaded an awkward dilemma, thanks to the assistance of the Y.M.C.A. and a number of sympathetic friends. In The Hospital on May 25 (p. 153) it was recorded that an attempt was being made to convert the chapel at their military hospital into a recreation-room for soldiers, and later, on June 22 (p. 245), that, thanks to the opposition of the Guardians, this foolish proposal was stopped by the War Council. The Guardians felt, however, that there was need for a recreation-room for the wounded soldiers, and wished to build one in the grounds, but on approaching the authorities for the necessary permission it was refused. They have now been informed that the Y.M.C.A. have agreed to erect a recreation-room and library in the grounds of the hospital, since the necessary subscriptions have been raised. The building will be erected entirely at the cost of the Y.M.C.A., but the Guardians may probably be required to instal fittings. This they have willingly decided to do, and to thank the Y.M.C.A. and the donors for their very kind gift.

### ARE HOSPITAL EXTENSIONS URGENT?

Glasgow Corporation proposes to extend its Knightswood Hospital, but the Ministry of National Service disposes, declaring that in view of the very serious shortage both of labour and materials it is only possible at the present time to consider the granting of licences for buildings of great national urgency. And so the Corporation is requested to consider postponing the application until the position in regard to labour and material may be less acute. The Health Committee of the Corporation concludes that the extension cannot be postponed, and suggests a conference with the Ministry on the subject.

### THE LATEST HOSPITAL FOR SHELL SHOCK.

On the first day of the month was opened the new home of recovery for neurasthenic sailors and soldiers at Bray Court, Maidenhead, which, as deputy for the Ministry of Pensions, is managed by the National Hospital for the Paralysed and Epileptic, Queen Square, and is the second of the group of hospitals to be opened or taken over, for the treatment of pensioners, by that famous hospital for nervous diseases. The fifty beds at Bray, which we understand will later be added to, will relieve the waiting-list of shell-shock and neurasthenic patients that have passed through the hands of the Special Medical Board at Lancaster Gate. The making of a hospital, even of fifty beds, is a matter of care and consideration, and the authorities at the National will now be able to take a breathing-space after passing the newly-decorated and newly-equipped hospital over to a carefully chosen staff, the heads of which are Dr. G. Stewart, the resident medical officer, and Miss Drowley, the matron-in-charge. There is a garden of some eighteen acres at Bray Court, and amongst the trades taught to the patients will be gardening in all its branches and poultry farming. These occupations, with carpentry and painting, will be the first chosen, and amidst the peaceful surroundings of the beautiful Thames-side locality, aided by all the possible adjuncts of science, the care and happiness of the patients should in most cases quickly result.

### THE INVALIDED CANADIAN SOLDIER.

When an invalided Canadian soldier is discharged he is given, in a handy form, the addresses of all the assistant directors of the Invalided Soldiers Commission, district officers of the Pensions Board, and secretaries of the Provincial Soldiers' Employment Commission. These addresses are also exhibited in every Post Office in Canada. It is made clear to the man that his pension, being for disability caused or aggravated by service, cannot be reduced by his taking work or perfecting himself in an industry, and that if his disability prevents him from taking up his old employment, free training for a new occupation will be provided for him. The Government of the Dominion are resolved that no man shall suffer from his disability more than can be avoided. It has been arranged that preference shall be given to such men in filling Government and municipal appointments.

### MEAT RATIONS AND MATERNITY.

Those who are in charge of maternity feeding centres should note that certain new regulations by the Food Controller have come into force. The maximum amount of meat which maternity feeding centres are now permitted to supply without requiring the surrender of a coupon is 8 oz. (including bacon and ham and all ration-free meat) per person per week. A $\frac{1}{4}$ lb. of sausages, or $\frac{1}{2}$ lb. of edible offal (excepting tongues, kidneys and ox-skirt) or 1 lb. of bones count, however, as 1 oz. of meat. Maternity feeding centres which undertake to confine consumption within these limits will be exempted from the requirement to keep the ordinary institution's register of consumption, but will be subject to certain special rules. These latter specify that only one meal a day must be served to any one person, and that the recipients must be expectant or nursing mothers only. The following scale is laid down for the consumption of other rationed foods:—For each meal served, $\frac{1}{5}$ oz. of butter or margarine, $\frac{1}{4}$ oz. of lard or edible fats, $\frac{1}{7}$ oz. of sugar. No sugar may be used for sweetening beverages. Feeding centres which prefer to serve more meat, or more than one meal a day, must collect coupons in the ordinary way as applied to institutions serving non-residents.

### THE COLLAPSE IN THE BALKANS AND THE DRUG SUPPLY.

The collapse of Bulgaria and its probable—if not inevitable—effect on Turkey and Austria-Hungary, and, more important still, on the duration of the war, are topics that are being discussed throughout the world to-day. But, every man to his trade, the druggist is already wondering how the Bulgarian *débâcle* is going to affect the drug supply. As a matter of fact Bulgaria is not an important drug-producing country; it is, of course, far famed for its attar of roses, which at the present time is worth more than its weight in gold. The stocks that have accumulated will presumably be released before long, and its value on the London market should drop like the proverbial rocket-stick. But this, indeed, is a small affair. What is far more important from the point of view of the medical man is that the collapse of Turkey, which is sure to follow the surrender of the Bulgarians, will liberate large supplies of opium and several other valuable drugs. During the past few years makers of morphine have been using Persian and Indian opium, and useful as these have been in an emergency, they are inferior to the Turkish variety for manufacturers' purposes. The Central Powers have, no doubt, been drawing extravagant supplies of opium from Turkey, but in spite of this she must still hold a large accumulation of stocks which, in view of the requirements of the Medical Services of our own and our Allies' armies, will be a booty not to be despised.

### THIS WEEK'S DRUG MARKET.

The market continues fairly active, but naturally, in view of the great events of the past week and the possibility of further developments in the near future, buyers are acting with caution. It would be unwise to take in any considerable stocks without making even fuller inquiries than usual about the position of every single drug it is contemplated to purchase. The time has now come when it is more important than ever to look ahead. The drugs that come from the Near East are not particularly numerous, but one of them—Turkey opium—is specially important. But in the event of a collapse of Austria-Hungary the source of a wide range of drugs could be reopened. Business has recently been done in quinine at advanced rates, but those who can afford to wait would not be ill-advised to do so, as supplies may be more plentiful before long. The values of menthol, Japanese peppermint oil, and lemon oil are maintained, but business in these articles has been less active. Japanese refined camphor is again dearer. Business has been done in honey at advanced prices, but the rumour is again current that the Ministry of Food intends to fix maximum selling prices.

